# Development of an Electronic Nose for Environmental Odour Monitoring

**DOI:** 10.3390/s121114363

**Published:** 2012-10-25

**Authors:** Licinia Dentoni, Laura Capelli, Selena Sironi, Renato Del Rosso, Sonia Zanetti, Matteo Della Torre

**Affiliations:** 1 Politecnico di Milano, Department of Chemistry, Materials and Chemical Engineering “Giulio Natta”, Piazza Leonardo da Vinci 32, 20133 Milano, Italy; E-Mails: laura.capelli@polimi.it (L.C.); selena.sironi@polimi.it (S.S.); renato.delrosso@polimi.it (R.D.R.); 2 Progress S.r.l., via N.A. Porpora 147, 20131 Milano, Italy; E-Mail: s.zanetti@olfattometria.com; 3 Sacmi s.c., via Provinciale Selice 17/a, 40026 Imola, Italy; E-Mail: Matteo.DellaTorre@sacmi.it

**Keywords:** odour detection, odour concentration, classification, field monitoring, humidity regulation

## Abstract

Exhaustive odour impact assessment should involve the evaluation of the impact of odours directly on citizens. For this purpose it might be useful to have an instrument capable of continuously monitoring ambient air quality, detecting the presence of odours and also recognizing their provenance. This paper discusses the laboratory and field tests conducted in order to evaluate the performance of a new electronic nose, specifically developed for monitoring environmental odours. The laboratory tests proved the instrument was able to discriminate between the different pure substances being tested, and to estimate the odour concentrations giving correlation indexes (R^2^) of 0.99 and errors below 15%. Finally, the experimental monitoring tests conducted in the field, allowed us to verify the effectiveness of this electronic nose for the continuous detection of odours in ambient air, proving its stability to variable atmospheric conditions and its capability to detect odour peaks.

## 1. Introduction

In recent years there has been a growing interest among the population and environmental protection authorities in issues related to the emission of odours and odorous substances from industrial activities [1,2]. As a consequence, several studies have been carried out in order to develop specific methodologies for monitoring air quality and evaluating nuisance odours [3].

Techniques for the measurement of odours and odorous substances are nowadays consolidated and widely used for the quantification of odour emissions at the emission source [4]. Such techniques include on the one hand instrumental detection (indirect methods), *i.e.*, the use of analytical techniques such as gas-chromatography coupled with mass spectrometry (GC-MS), which allows the identification and quantification of the odorous compounds of an emission. One drawback of this approach is that, especially when dealing with complex odours, it may be difficult to relate the chemical composition of an odorous mixture to the odorous sensation provoked by the mixture on humans [5,6]. The effects of odorant mixing are often reported to be highly complicated, producing: (i) averaging [7], (ii) hypoadditivity (lower than the sum or average) [8,9], (iii) masking [10], and (iv) synergistic effects [11,12]. On the other hand, direct methods, *i.e.*, sensorial techniques using the human nose as a sensor, thus enabling characterization of odours by referring directly on their effects on a panel of qualified examiners, are more and more often used for odour impact assessment purposes. Among these, dynamic olfactometry is the most common one. Dynamic olfactometry is a sensorial technique which allows determination of the so called odour concentration, which is measured by determining the dilution factor required to reach the detection threshold and expressed in odour units per cubic metre (ou_E_/m^3^). The odour concentration at the detection threshold is by definition 1 ou_E_/m^3^, giving that the odour concentration of an odorous sample is expressed in terms of multiples of the detection threshold. This technique has been recently standardized by the European Norm EN 13725:2003 [13], which contributed to partially overcome the problems due to the variability of human olfaction between different subjects, making the olfactometric measurements results more reliable and repeatable [14,15]. Still, the use of olfactometry entails the limitation of solely quantifying odours in terms of intensity or concentration, without giving any information about odour quality [16].

However, besides source characterization, exhaustive odour impact assessment should involve the evaluation of the impact of odours directly on citizens. The measurement of odours at receptors, *i.e.*, at a far distance from the source and therefore at very low concentrations, involves several technical difficulties, for which techniques for the quantification or identification of odours in ambient air are not well defined and consolidated as those applied for source characterization [17].

Odour impact assessment at receptors should entail the quantification of the presence of odours, e.g., the time frequency with which odour is perceived or a given odour concentration is exceeded. Moreover, where more industrial activities co-exist, the identification of the cause of the odour nuisance could be required [18]. For this purpose, it would be useful to have an instrument capable of continuously monitoring ambient air quality, detecting the presence of odours and also recognizing their provenance by attributing the analyzed air to a specific emission source [19].

Electronic noses can be used for this purpose. If properly trained, electronic noses can detect the presence of odours in ambient air, estimate odour concentration and attribute the perceived odour to a specific odour source [20]. During the last decade research activity aiming at the development of specific electronic noses for the continuous monitoring of environmental odours has been carried out at the Politecnico di Milano, in collaboration with Sacmi s.c. and Progress S.r.l.

Since the first instrument developed mainly for laboratory use (EOS 835) [21], during the last years an innovative electronic nose was realized (EOS 507) [22], designed with the aim of guaranteeing better performance in the field under variable meteorological conditions and with diluted odours [23].

This paper discusses the laboratory and field tests conducted in order to evaluate the performance of this new instrument. The performance evaluation in the laboratory was concerned specifically the verification of the ability to discriminate and correctly classify different specific odorous compounds (pure substances selected as representative of typical environmental odour emissions) and to estimate their odour concentration.

The electronic nose performance was further verified by a field monitoring trial conducted in a rural area in the north of Italy where three odour emitting plants are present, with the aim of identifying the major source of annoyance. During the monitoring four electronic noses EOS 507 were used together with an “old” EOS 835 electronic nose, in order to compare the instrument performances and thus verify the effectiveness of the innovations and improvements introduced in the new EOS 507.

## 2. State of the Art

One of the first studies published concerning the use of an electronic nose to monitor the presence of different odours in ambient air was performed by Misselbrook *et al.*[24]. In their work the authors compared the reliability of two different devices, Odourmapper (developed by UMIST) and Aromascan (Aromascan plc, Crewe, UK) in odour quantification. Sensor responses of both instruments to different samples with variable odour concentration had the same trend, but variances of the experimental data responses were not satisfying (62 and 59%, respectively). A better correlation between odour concentration and sensors responses was found by Stuetz *et al.*[6]. The first works on odour quantification performed by electronic noses showed that the algorithms used for odour concentration estimation and the investigated odour concentration range are crucial factors that can affect the analysis. Micone and Guy [25] obtained good reliability by using an electronic nose for analyzing odour samples with an odour concentration between 50 and 150 ou_E_/m^3^. However, the instrument accuracy turned out to worsen when the odour concentration increased.

Another important factor to be considered is the influence of environmental conditions on sensor responses. As a matter of fact, the previously reported results were obtained in laboratory tests, *i.e.*, in an enclosed environment with almost constant and controlled conditions. The use of electronic noses in the field is more challenging, due to variable weather conditions, and especially due to variations in atmospheric temperature and humidity content [26]. As a consequence, electronic noses have to minimize or compensate the effects of such variations in order to produce reliable results. In recent years, several studies were performed in order to develop an electronic nose to be used in the field. Nicolas *et al.*[27] developed an instrument to be used in the field and, after a proper training, used it to detect and classify odours near facilities having different odour emissions, such as a waste water treatment plant, a composting plant, a paint house and a sugar factory. Tests proved the instrument to be able to distinguish between the different olfactory classes, but such an ability was influenced by atmospheric conditions and by the sensor response drift over time. In another study, the same authors [28], verified the capability of an electronic nose to distinguish between different odours from a composting plant. In the following years, Sironi *et al.*[29] performed tests to verify the possibility of using the electronic nose they developed to classify odorous ambient air. A monitoring trial with a duration of 4 days was performed and results were compared with odour reports from inhabitants, obtaining a correspondence of 72%. Another monitoring was performed by Sironi *et al.* in 2007 [30] to evaluate which features had to be extracted from the sensor response curves in order to optimize the correlation between odour classification performed by an electronic nose and odour complaints from inhabitants. During this study, the variability of humidity content in analyzed air turned out to be a critical factor in the use of electronic noses for environmental air monitoring.

The influence of feature extraction on odour classification is discussed also by Sohn *et al.*[20]. This study also shows that the accuracy of the classification increased when the number of olfactory classes considered for the classification was reduced.

Among the factors affecting the classification accuracy, Romain *et al.*[31] studied the influence of sensor drift, reporting that the classification accuracy decreased with the passage of time from 98% at the beginning of the tests to 20% after four years. In order to avoid this accuracy decrease authors suggest training the electronic noses repeatedly during the monitoring period.

Another approach to solve the problem is presented by Capelli *et al.*[22] who developed an electronic nose with an internal calibration system that allows one to evaluate sensor drift daily and to compensate it with suitable algorithms. Moreover, the electronic nose is equipped with a specific humidity regulator that allows the instrument to be used in the field under variable meteorological conditions.

## 3. Materials and Methods

### 3.1. Employed Electronic Noses

**Figure 1.**
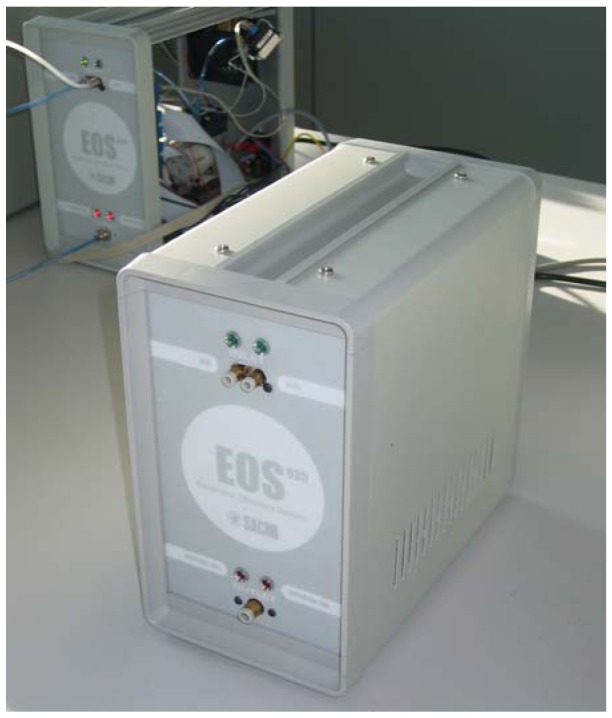
The EOS 835 electronic nose.

**Figure 2.**
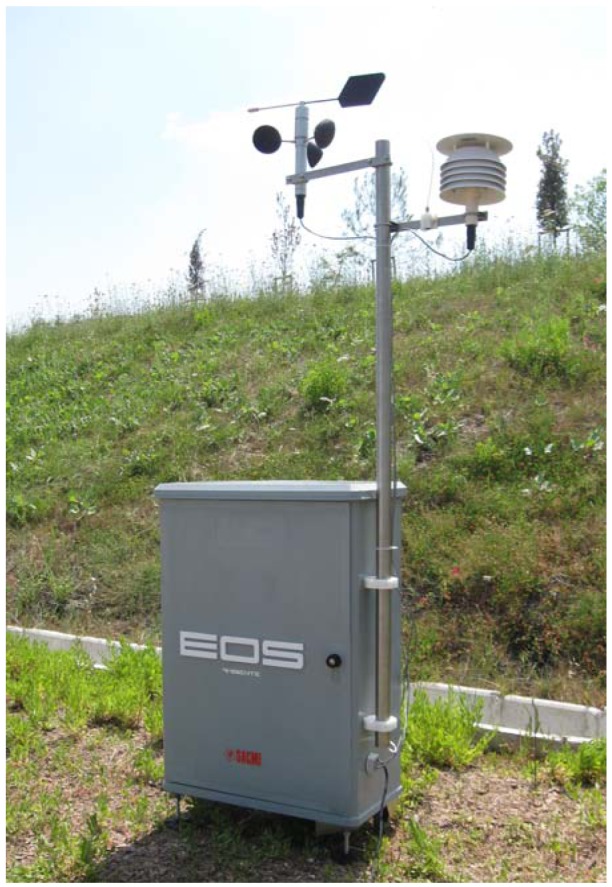
The EOS 507 electronic nose.

Two different kinds of instruments were used for this study, *i.e.*, the “older” EOS 835, and the new EOS 507. The EOS 835 [21,32] (Figure 1) is an electronic nose that was first designed for laboratory use, and it represented the starting point for the development of the new, specific electronic nose to be used in the field for the continuous monitoring of environmental odours, *i.e.*, the EOS 507 (Figure 2). Both kinds of electronic noses are equipped with six MOS sensors, which respond to the presence of odorous compounds in the analyzed air by changing their resistance with respect to specific reference conditions [33]. Currently, in most commercial electronic noses (including the EOS 835) the reference conditions are obtained by fluxing neutral air (*i.e.*, clean, non-odorous air, obtained for instance by filtration through activated carbon or other neutral air generation devices). The resistance variation towards the reference produces a response curve, from which significant features can be extracted for further numerical elaborations and classification.

The EOS 835 electronic nose has two air inlets: the first is connected to a filter with active carbon and silica gel for the realization of the reference “neutral air”, whereas the second (“sample air” line) is connected to a valve regulating the sample air flow directed to the sensor chamber. During the “cleaning”, or reference phase, “neutral air” flows over the sensors. During measurement, the inlet is switched to the sample air, the composition of the analyzed mixture changes, and the sensor resistances change correspondingly, thereby generating a response curve for each sensor.

The training phase requires the analysis of suitable odour samples which should be representative of the odours the electronic nose will be required to recognize during the subsequent monitoring period. After collection on the odour sources to be considered for the monitoring, samples are analyzed by dynamic olfactometry for determination of odour concentration, and finally diluted at a suitable concentration range, which previous research has proved to be comprised between 100 and 200 ou_E_/m^3^[34]. Among the olfactory classes to be considered for electronic nose training, “neutral air” (*i.e.*, non-odorous air) shall be included as well.

During the monitoring period, the EOS 835 electronic nose analyzes the air for three consecutive minutes out of 15 (3 minutes analysis +12 minutes cleaning). At the end of the monitoring period the collected data must be processed in order to extract significant features from the sensor response curves (e.g., the difference between the sensor resistance in specific points of the curve compared with the reference conditions, or the area subtended by the response curve) to be used for odour recognition.

In order to optimize the sample air classification a feature selection has to be performed, as to select the features accounting for the best discrimination of the considered olfactory classes [30]. This operation is performed using multivariate data analysis techniques such as cross validation and PCA [35]. Once the features are selected, the instrument performs the classification of the unknown measures using a KNN algorithm [36].

The EOS507 electronic nose has some innovative aspects with respect to its forerunner EOS 835 and to other currently available electronic noses. The main innovations were introduced in order to minimize the influence of the atmospheric conditions and of the sensor drift on the field measurements.

First, the instrument is equipped with a system for the adjustment of the sample air humidity to a fixed value, calculated to optimize the instrument regulation capability based on the measured external ambient air humidity.

**Figure 3.**
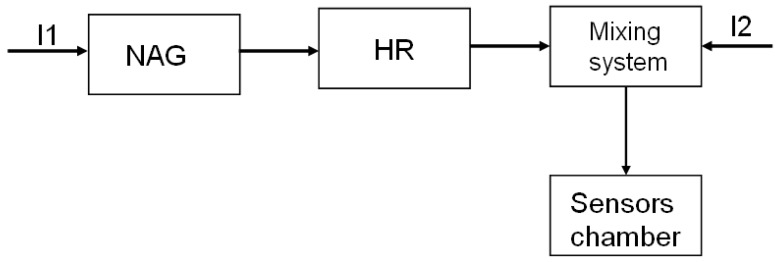
Scheme of the EOS 507 airflows.

The instrument has two inlets for the ambient air. As shown in Figure 3, the first inlet (I1) is connected to a system for the realization of “neutral air” (Neutral Air Generator—NAG), *i.e.*, air that doesn't cause a variation in the sensor resistance. This neutral air stream is drawn to the humidity regulator (HR) that regulates the humidity of the neutral air to bring the humidity of the final mixture that is fluxed into the sensor chamber to a fixed value.

This value is constant within a measurement session but may be changed in function of the external conditions (*i.e.*, the external ambient humidity), to keep the humidity regulator in its optimal working range. The humidity regulation of the neutral air stream I1 is sufficient to guarantee the desired final humidity value, thereby entailing the advantage of not varying directly the humidity content of the sample air (I2), thus avoiding the risk of interfering with its composition.

A second important innovation concerns the use of a reference which is not neutral air, but a specific substance at known and constant concentration, called “standard”. This has the advantage of producing a more stable reference baseline, as little impurities in the “standard” flow do not produce considerable oscillations of sensors resistance as it occurs using neutral air as a reference.

The use of this “standard”, different from neutral air, drastically changes the functioning principle of this electronic nose with respect to its precursor EOS 835 or to other similar instruments. Indeed, its functioning is based on the periodic analysis of the “standard”, *i.e.*, the reference substance at a fixed concentration, which serves as periodic re-calibration of the system: sensor responses are normalized to the “standard”, thus compensating sensor drift over time. The “standard” phase also has a similar function as the cleaning phase in other electronic noses, because, as a matter of fact, it brings back the sensor responses to a baseline, represented by their response towards the “standard”. In order to prevent sensor poisoning, and to optimize the electronic nose performance in terms of repeatability, the “standard” phase shall be carried out rather frequently. Actually, the standard phase is carried out daily, and has a duration of about 2 hours.

Given the different functioning principle of the new EOS 507, also the feature extraction operation of the sensor responses is different too. In this case, the response processing involves the extraction of just one feature for each sensor, defined as “Eos Unit” (E.U.), which is calculated by normalizing the sensor resistance during the measure with respect to the “standard” (reference) phase. The system performs the recognition of unknown measures referring to a database acquired in a previous training phase (training measures). In analogy with the EOS 835, both samples of neutral air and of the odours to be recognized have to be analyzed for electronic nose training.

Another innovative aspect of the EOS 507 consists in the calculation of a so called “threshold”, calculated based on the E.U. relevant to the neutral air measures, below which classification does not take place. In other words, when analyzing unknown samples, if the resulting E.U. are higher than the “threshold”, they are classified using a KNN algorithm [36]. Otherwise, the samples are classified directly as “neutral air”. Finally, the EOS 507 has an internal system for the automatic dilution of the samples used for the instrument training. This allows one to dilute the original sample at different dilution ratios, thus obtaining a concentration scale ranging from 10% to 100% of the original samples without requiring manual dilution and the preparation of more samples (bags) at the different concentrations to be analyzed. As far as the monitoring phase is concerned, the air is analyzed continuously and data are recorded once per second.

### 3.2. Laboratory Tests

#### 3.2.1. Aims

The laboratory tests had the aim to verify the capability of the new instrument (EOS 507) to discriminate between odour samples of pure compounds, chosen as to be representative of industrial odour emissions. Preliminary tests were run in order to evaluate the possibility of quantifying odour, as well.

#### 3.2.2. Tested Compounds

The compounds to be tested were selected among typical compounds that can be found in environmental odour emissions, thereby considering compounds including different functional groups. The compounds used were limonene, ethanol and dimethyl sulfide. The mixtures to be analyzed were prepared from the liquid compounds, by inserting the liquid into a sampling bag and then filling the bag with neutral air. The obtained samples were stored at fixed temperature and humidity, in order to guarantee measurement repeatability, and then analyzed by dynamic olfactometry for odour concentration determination.

#### 3.2.3. Test Method

The laboratory tests were carried out in order to verify the reliability of the new electronic nose (EOS 507) in classifying odours and in quantifying them. Therefore the EOS 507 electronic nose was trained with mixtures of pure compounds as well as with neutral air. As previously mentioned, the electronic nose has an internal automatic dilution system of the training samples. For this reason, samples of pure compounds were prepared and diluted to obtain samples having an odour concentration of about 300 ou_E_/m^3^, which was chosen in order to obtain a concentration scale ranging from 30 ou_E_/m^3^ (10% of the original sample) to 300 ou_E_/m^3^ (100%), thus including the above mentioned “optimal” odour concentration range comprised between 100 ou_E_/m^3^ and 200 ou_E_/m^3^[34]. The measurements were conducted in triplicate for each compound.

Neutral air samples were analyzed in order to determine the E.U. relevant to neutral air required for the calculation of the above mentioned “threshold”. In order to evaluate the instrument capability to discriminate the different odours tested the experimental data were analyzed by PCA.

Then, the instrument capability to estimate odour concentration was assessed by performing analyses with specific samples prepared at different known concentrations. The electronic nose was used to estimate their odour concentration, and the quantification performance was evaluated by comparing the values measured by electronic nose *vs.* true values measured by dynamic olfactometry.

### 3.3. Field Test

In order to verify the effectiveness of the innovations introduced in the EOS 507 with respect to the EOS 835, a monitoring in the field using both kinds of instruments was performed. More in detail, four EOS 507 and one EOS 835, each equipped with six MOS sensors (Table 1), were used.

**Figure 4.**
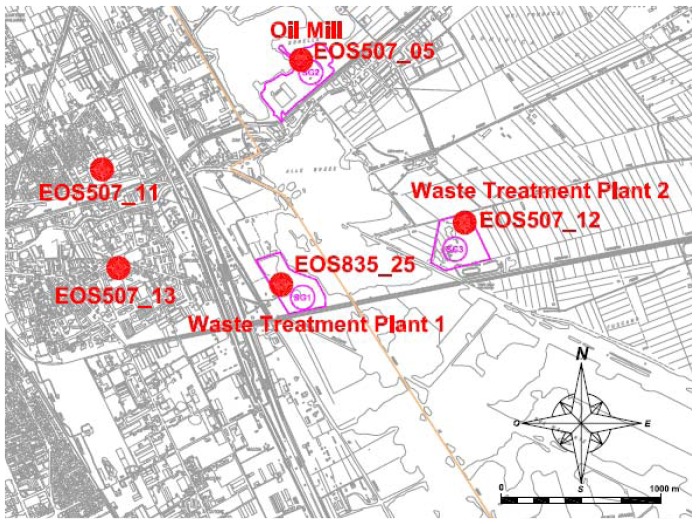
Position of the electronic noses.

EOS 507_05, EOS 507_12 and EOS 835_25 were positioned at the emissive plants' boundaries, whereas EOS 507_11 and EOS 507_13 were placed in the surrounding urban area at receptors where complaints were reported (Figure 4).

The instruments were employed in order to determine the odour exposure in ambient air in an area where several industrial activities are present. More in detail, an oil mill and two waste treatment plants were considered as possible odour sources. An olfactometric campaign was performed in order to identify the principal odour sources and to collect odour samples directly at the emission sources, to be analyzed by dynamic olfactometry for the determination of the odour concentration. The odour concentration measurement is useful both to evaluate the emission entity and to determine the dilution factor to be applied for obtaining suitable samples for electronic nose training. In order to increase training data robustness, the olfactometric campaigns for the collection of samples for the electronic nose training were repeated in two different days, and the training operations took a whole week time.

The quality of the training data as well as the capability of the electronic noses of correctly classifying unknown measures was evaluated by performing cross validation. After the training phase, the electronic noses were positioned in the field (Figure 4) for a 10-day period. The data collected by the electronic noses were opportunely processed and, together with the meteorological data relevant to the monitoring period, analyzed in order to determine the source of nuisance odours in the monitored zone.

## 4. Results and Discussion

### 4.1. Laboratory Tests

**Figure 5.**
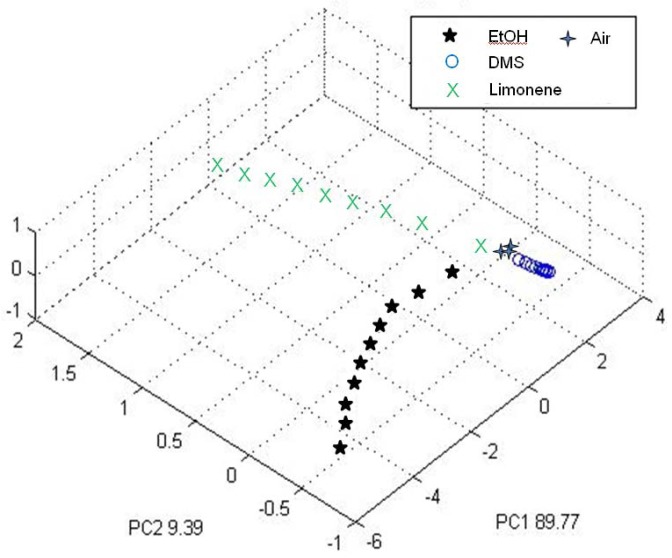
PCA of the laboratory tests measures performed with EOS 507.

The measures relevant to the training phase of the EOS 507 electronic nose using samples of pure compounds at different concentrations (in a range from about 30 to 300 ou_E_/m^3^) were analyzed by PCA. Figure 5 reports the results of PCA analysis considering the first three principal components, whereby the numbers reported near the axes indicate the variance explained by each principal component (89.77% for PC1 and 9.39% for PC2, respectively). PC3 was added because it allows a better visualization of data discrimination.

This analysis proved the EOS 507 could effectively discriminate the samples containing the three different compounds, showing that the points relevant to the different compounds tested are located in different regions of the graph. Moreover, it is possible to observe a trend related to the sample concentration: more diluted samples are near to the measure of neutral air, whereas the distance from the neutral air increases with concentration.

Specific tests were run in order to verify the electronic nose's capability to quantify odours by estimating the odour concentration of samples having an odour concentration in a range between about 50 ou_E_/m^3^ and 360 ou_E_/m^3^. These tests were limited to limonene and ethanol, because the instruments turned out to be barely sensitive to dimethyl sulfide. The scarce sensitivity towards DMS is visible from the above discussed PCA, whereby the points relevant to the DMS at the different concentrations are all close to each other, thus indicating a low detection capability. Table 2 reports the results of the odour concentration determinations, as well as the per cent error calculated as:

(1) $$\text{Err}(\%)=\frac{c_{od,EN}-c_{od,\text{olf}}}{c_{od,\text{olf}}}$$

where *c_od,EN_* and *c_od,olf_* are the odour concentrations measured by electronic nose and by dynamic olfactometry, respectively.

**Figure 6.**
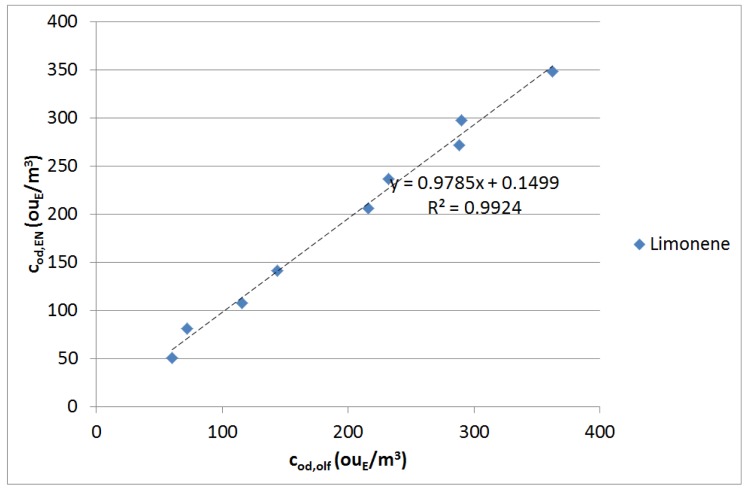
Estimated *vs.* real odour concentration of the limonene samples.

**Figure 7.**
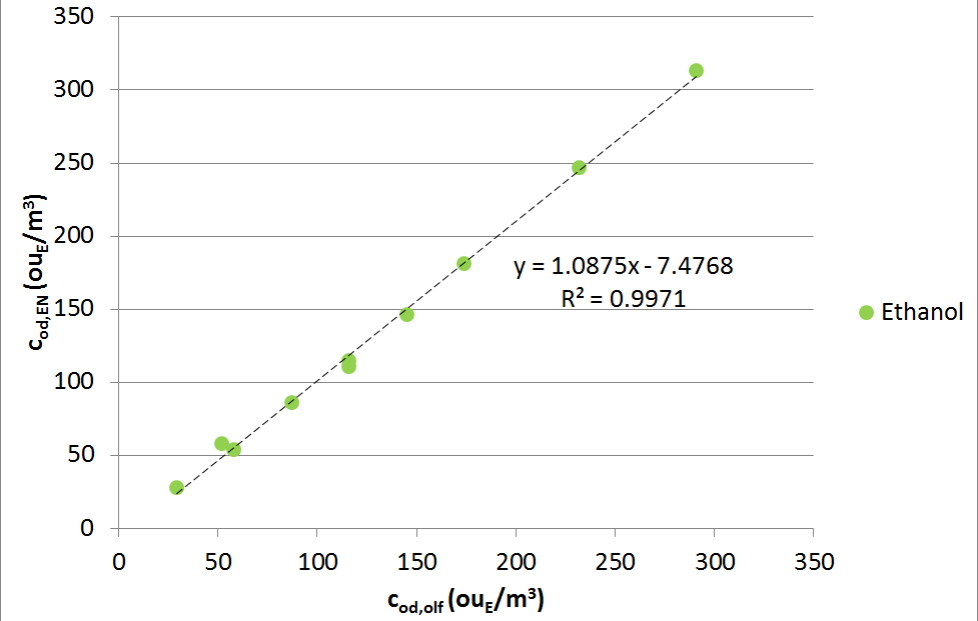
Estimated *vs.* real odour concentration of the ethanol samples.

Figures 6 and 7 illustrate the correlation between the odour concentrations estimated by electronic nose and those measured by dynamic olfactometry for the limonene and ethanol samples, respectively. Two data sequences are visible for each compound, because measures were repeated in two different days in order to verify repeatability and reproducibility of results. Repeated laboratory tests conducted on the same compound proved the new EOS 507 electronic nose's repeatability to be around 20%.

The test results show a very good correlation between the odour concentrations values, giving an average per cent error of 6.1% (maximum 15%) for limonene and of 4.7% (maximum 11.5%) for ethanol, respectively, and correlation indexes (R^2^) in both cases over 0.99.

These results seem promising, and further experiments will be run for verifying the instrument capability of accurately estimating the odour concentration of extremely diluted samples (*i.e.*, low odour concentrations) as well as for evaluating the influence of the training typology on the quality of the estimation.

### 4.2. Field Tests

#### 4.2.1. Monitoring Results

The electronic noses were placed in the field for a period of 10 days. The main purpose of the monitoring was to identify the major sources of odours perceived at receptors. For this reason, the monitoring results are expressed, for each of the electronic noses used, as the percentage of measures classified as coming from each plant being considered. The EOS 507 electronic noses have the possibility of classifying the analyzed air as “Unknown” when the instrument perceives odour, but the odour footprint is different from the odour footprints of the olfactory classes considered for training. The monitoring results are reported in Table 3, where the abbreviations WTP 1 and WTP 2 stand for the first and the second waste treatment plants monitored, respectively (Figure 4).

Based on the monitoring results reported in Table 2 it is not possible to identify the plant that mostly contributes to odour nuisance in the monitored zone. All electronic noses used for the monitoring attributed the majority of the measures to the olfactory class “Neutral air”.

As predictable, the electronic noses installed at the monitored plants' boundaries (*i.e*., EOS 835_25, EOS 507_05 and EOS 507_12, installed at the waste treatment plant 1 (WTP 1), the oil mill and the waste treatment plant 2 (WTP 2), respectively) attributed a significant percentage of measures (17.2% for EOS 835_25, 6.1% for EOS 507_05 and 6.8% for EOS 507_12, respectively) to the olfactory class corresponding to the plant they were located at.

Indeed, the two electronic noses placed at the receptors (*i.e.*, EOS 507_11 and EOS 507_13) rarely attributed the detected odours to one of the monitored plants, instead, the majority of the odour episodes were classified as “unknown”. This might indicate the presence, in the studied area, of other sources of odour nuisance than the ones considered during the training phase.

**Figure 8.**
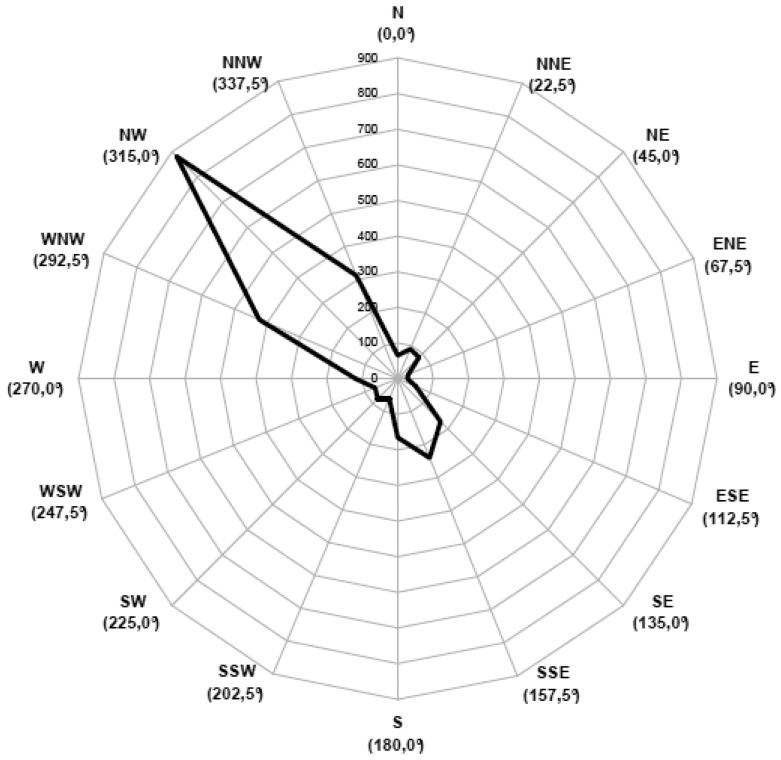
Wind rose relevant to the monitoring period (the wind rose indicates the wind direction vectors).

Moreover, the meteorological conditions of the monitoring period, for which the prevailing wind direction was from South-East to North-West (Figure 8), did not enhance the diffusion of the emitted odours towards the receptors where the electronic noses were installed (Figure 4). An analysis of results focused on the periods when the receptors were downwind with respect to the plants is rather complicated, due to the complex relative locations of the different monitoring points. Nonetheless, a raw restriction of the results to the periods when the wind blew from North-East, East and South-East still shows a similar trend (major percentage of measures attributed to “neutral air” followed by “unknown”), thus supporting the hypothesis of the presence of another, not considered, source of odour.

#### 4.2.2. Comparison EOS 507 *vs.* EOS 835

It is possible to make some general considerations about the functioning of the different instruments used for the study.

First, as far as the instrument training is concerned, the possibility of analyzing samples at different concentrations with the automatic dilution system of the EOS 507 significantly reduces the sample preparation times and is therefore very useful from the operational point of view.

As far as the features to be extracted from the sensor responses are concerned, the EOS 835 requires the calculation of several features and a feature selection for recognition optimization. This operation, which has to be done by an operator, makes the entire recognition process more complicated and subject to the operator's decisions.

Instead, the EOS 507 calculates only one feature for each sensor and directly performs the recognition of the sample air, thus reducing the times required for data processing and the influence of the operator's choices on the recognitions.

Regarding the monitoring phase, an important difference is given by the frequency of the analyses: the EOS 835 performs a measurement every 15 minutes, whereas the EOS 507 performs one recording per second.

**Figure 9.**
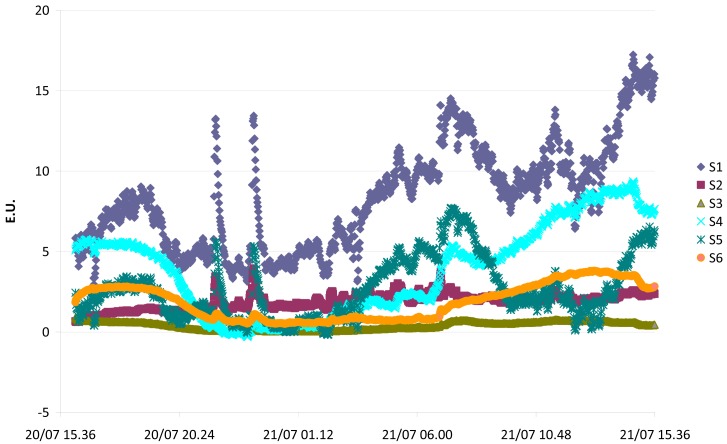
Plot of the EOS 507_05 monitoring data (E.U.) for the six sensors (named S1-S6 in Table 1).

The monitoring data obtained from the EOS 507 can be plotted in graphs in which the values of the features calculated for each sensor over time are represented. Figure 9 is shown as an example. These kinds of plots are interesting, because they allow an easy and immediate identification of “peaks” in sensors responses, *i.e.*, of periods corresponding to potential odorous events.

**Figure 10.**
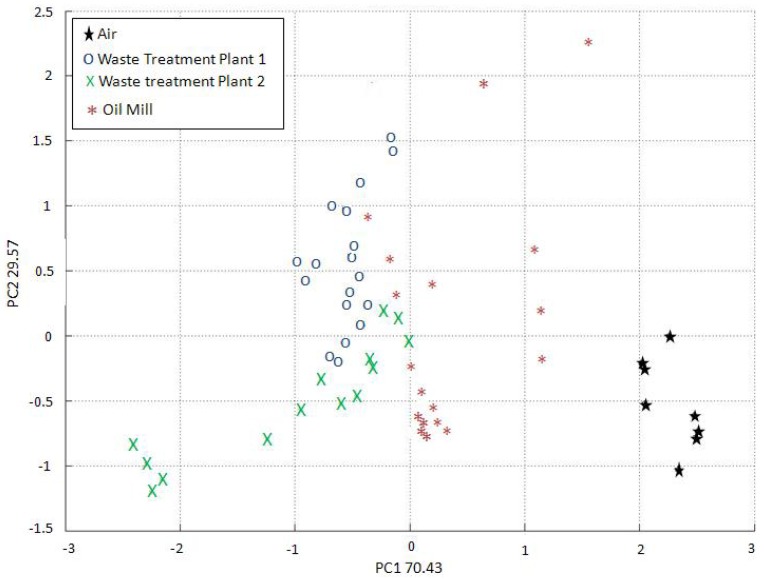
PCA of the EOS 835 training data.

**Figure 11.**
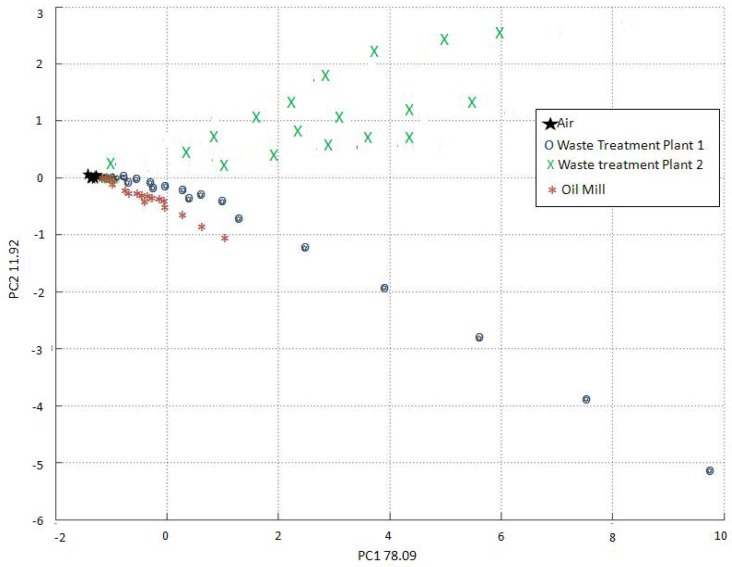
PCA of the EOS 507 training data.

The EOS 835 does not include the option to classify the measures as “unknown”, and is therefore forced to attribute any unknown measure to one of the olfactory classes considered during training. The EOS 507 has therefore improved the reliability of the recognition procedure. In order to evaluate the recognition performances of the two instruments, the training data relevant to both the EOS 835 and the EOS 507 were analyzed by PCA (Figures 10 and 11, respectively).

The reported PCAs are very different, due to the different training methods required by the two electronic nose typologies. The EOS 835 electronic noses had to be trained with samples having a fixed concentration in a range between 100 and 200 ou_E_/m^3^, whereas the EOS 507 electronic nose could be trained with a sample having a higher odour concentration (500 ou_E_/m^3^), as the instrument has the possibility of automatically diluting the original sample from 10% to 100%, as previously described in Section 3.1.

The PCA clustering relevant to the EOS 835 measurements is satisfying, but the distinction visible in Figure 11 is more clear, thus proving the EOS 507 to be more effective in odour discrimination.

Besides a qualitative analysis by PCA, the training data were further evaluated by means of cross validation. The classification error obtained with the two different electronic nose typologies, expressed as percentage of misclassified measure with respect to the total number of measures considered, are reported in Table 4.

The observations resulting from this study prove that the innovations introduced in the EOS 507 improve the instrument performance in terms of ease of use and odour discrimination capability with respect to the old EOS 835, thus making it more suitable for the continuous monitoring of environmental odours.

## 5. Conclusions

Laboratory and field tests reported herein were carried out to evaluate the performance of the new EOS 507 electronic nose, which has been specifically developed for the continuous monitoring of environmental odours. The laboratory tests conducted with odour samples prepared using pure substances proved the new instrument to be able to discriminate the different odours being tested, and to estimate the odour concentration of samples between about 50 ou_E_/m^3^ and 300 ou_E_/m^3^, giving correlation indexes (R^2^) of 0.99 and errors below 15%.

A field test, consisting in a 10-day monitoring of an area where different odour emitting plants are present, was then conducted to verify the effectiveness of the innovations introduced in the EOS 507 with respect to the EOS 835.

The new electronic nose guarantees a better clustering of training data as can be deducted by the PCA plots and, as a consequence, a more reliable classification of the perceived odours. Moreover, the EOS 507 electronic nose is more user-friendly, due to the presence of specific devices that reduce the need of operator actions. In addition, the reduction of the features that are calculated from the sensors responses avoid the degrees of freedom in data processing, thus guaranteeing a higher robustness of results.

Finally, the E.U. plot that can be derived from the EOS 507 sensors responses is useful as it allows an easy and immediate identification of possible odour peaks.

## Figures and Tables

**Table 1.** Sensor arrays of the five electronic noses employed for the field test.

	**EOS 835_25**	**EOS 507_05**	**EOS 507_12**	**EOS 507_11**	**EOS 507_13**
S1	SnO_2_ + SiO_2_	SnO_2_ + Mo	SnO_2_ + Mo	SnO_2_ + Mo	SnO_2_ + Mo
S2	SnO_2_	SnO_2_	SnO_2_	SnO_2_	SnO_2_
S3	SnO_2_ + Mo	SnO_2_ + MoO_2_	SnO_2_ + MoO_2_	SnO_2_ + MoO_2_	SnO_2_ + MoO_2_
S4	SnO_2_ + Au	SnO_2_ + WO_3_	SnO_2_ + WO_3_	SnO_2_ + WO_3_	SnO_2_ + SiO_2_
S5	SnO_2_	SnO_2_	SnO_2_	SnO_2_	SnO2
S6	SnO_2_ + In_2_O_3_	SnO_2_ + TiO_2_ + NbO_5_	SnO_2_ + TiO_2_ + NbO_5_	SnO_2_ + TiO_2_ + NbO_5_	SnO_2_ + TiO_2_ + NbO_5_

**Table 2.** Odour concentration values estimated by electronic nose (c_od,EN_) *vs.* measured by dynamic olfactometry (c_od,olf_) and per cent estimation error relevant to the limonene and ethanol samples.

**Compound**	**c_od,olf_(ou_E_/m^3^)**	**c_od,EN_(ou_E_/m^3^)**	**Error (%)**
Limonene	60	51	-15
	116	108	-6,9
	232	237	2,2
	290	298	2,8
	72	81	12,5
	144	141	-2,1
	216	206	-4,6
	288	272	-5,6
	362	349	-3,6

Ethanol	29	28	-3,4
	52	58	11,5
	87	86	-1,1
	116	115	-0,9
	145	146	0,7
	58	54	-6,9
	116	111	-4,3
	174	181	4
	232	247	6,5
	291	313	7,6

**Table 3.** Percentage of measures attributed to the different olfactory classes during the monitoring period.

**Air quality**	**EOS 835_25 Measures (%)**	**EOS 507_05 Measures (%)**	**EOS 507_12 Measures (%)**	**EOS 507_11 Measures (%)**	**EOS 507_13 Measures (%)**
Neutral Air	80,6%	90,0%	91,9%	72,7%	70,9%
Unknown	--	2,2%	0,3%	24,6%	25,5%
Oil Mill	1,6%	6,1%	0,8%	1,4%	2,2%
WTP 1	17,2%	0,4%	0,2%	0,9%	0,2%
WTP 2	0,6%	1,3%	6,8%	0,4%	1,2%

**Table 4.** % of misclassified measures obtained by cross validation of the training data.

**Electronic nose**	**% of misclassified measures**
EOS 507	12%
EOS 835	18%
